# Enterovirus D68 Sequence Variations and Pathogenicity: A Review

**DOI:** 10.3390/v18010073

**Published:** 2026-01-04

**Authors:** Yi Zhu, Liting Wang, Jun Shen

**Affiliations:** Infectious Disease Department, Children’s Hospital of Fudan University, National Children’s Medical Center, Shanghai 201102, China; 23211240031@m.fudan.edu.cn (Y.Z.); 24211240020@m.fudan.edu.cn (L.W.)

**Keywords:** Enterovirus D68, pathogenicity, genomic variation, structural proteins, non-structural proteins

## Abstract

Enterovirus D68 (EV-D68), a neurotropic respiratory pathogen, poses a considerable clinical threat through its link to pediatric acute flaccid myelitis (AFM) and severe respiratory illness. The possibility of recurrent epidemics, evidenced since the 2014 outbreak, remains a major concern. Genomic determinants of virulence are central to this threat. Sequence variations that affect host–receptor interactions, immune evasion, and replication efficiency serve as critical modifiers of pathogenicity. This article systematically reviews the evidence for specific genomic sites that enhance EV-D68 virulence, focusing on three critical regions: the VP1 receptor-binding site, the 2Apro/TRAF3 cleavage site, and the 3Cpro immunoregulatory region. Mutations in the VP1 receptor-binding site can alter affinity for host receptors such as sialic acid, heparan sulfate, and MFSD6, thereby shaping viral entry and tissue tropism. Alterations in the 2Apro/TRAF3 cleavage site may impair proteolytic cleavage of host TRAF3, attenuating immune evasion and reducing viral pathogenicity. Variations in the 3Cpro region affect its efficiency in cleaving host proteins involved in translation and autophagy, ultimately modulating viral replication and antiviral responses. Finally, we propose that monitoring for mutations in these key virulence determinants, particularly within the surface-exposed VP1, is essential for effective outbreak preparedness.

## 1. Introduction

Enterovirus D68 (EV-D68), classified within the genus Enterovirus of the family Picornaviridae, was first isolated in 1962 from pediatric patients presenting with mild respiratory illness in California, United States [1,2]. In the subsequent decades, EV-D68 was largely regarded as a virus of low pathogenic concern, predominantly linked to subclinical infection or mild upper respiratory tract infections, such as rhinitis and pharyngitis, which symptomatology often overlapped with that of the common cold [2]. Seroepidemiological evidence indicates that children represent the primary reservoir for establishing population-wide immunity against EV-D68. Although adults remain susceptible to infection, pre-existing immunity and a more effective immune response typically confine EV-D68 infection to subclinical or mild presentations. This perception shifted markedly in 2014, when a widespread outbreak of EV-D68 across North America resulted in more than one thousand laboratory-confirmed cases, particularly among children, manifesting as severe respiratory diseases and a previously underrecognized neurological complication: acute flaccid myelitis (AFM), a polio-like syndrome characterized by the acute onset of limb weakness and paralysis [3,4,5]. This outbreak underscored the latent virulence of EV-D68 and prompted heightened scientific attention to its evolutionary dynamics and pathogenic mechanisms.

**Figure 1:**
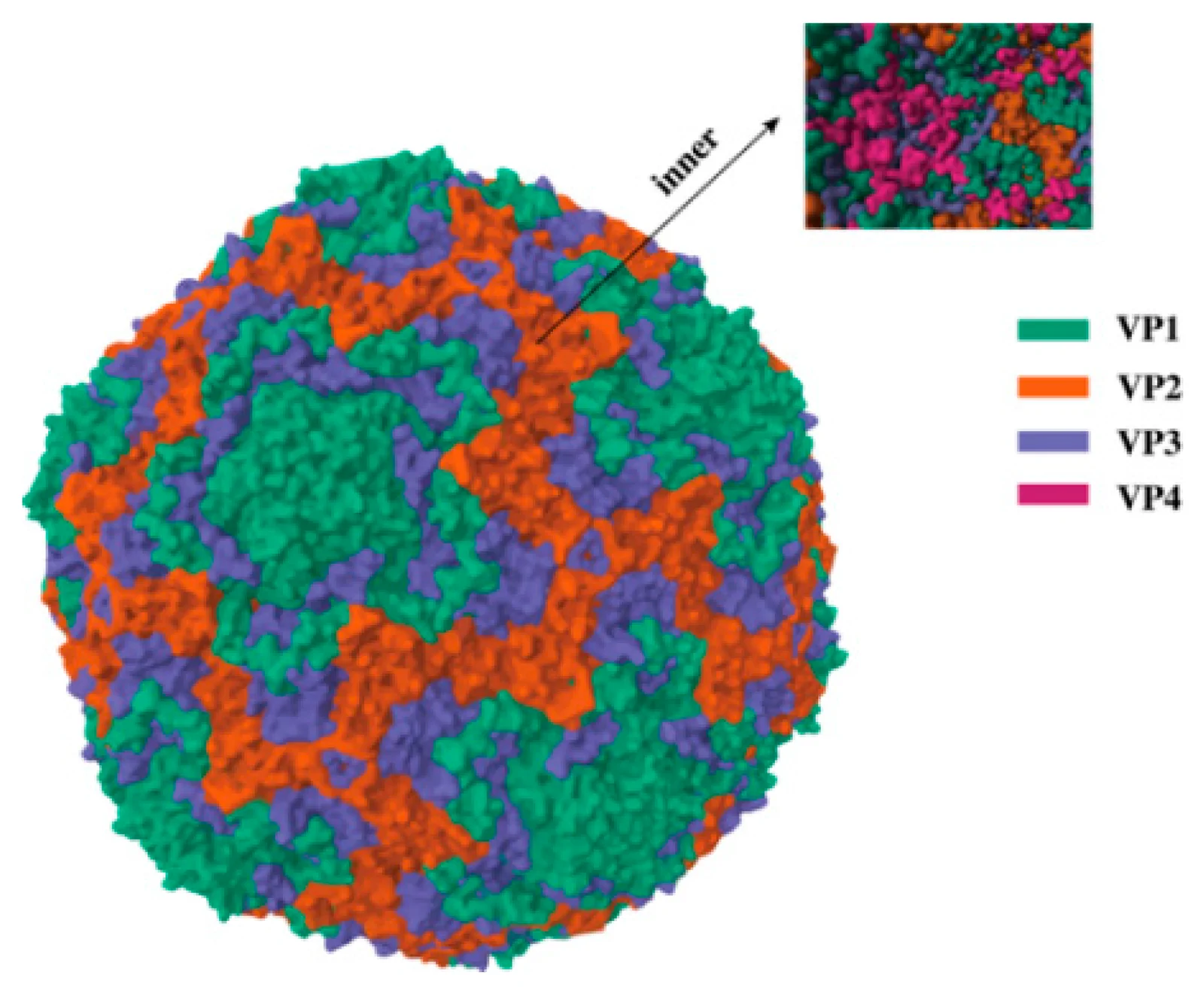
Structural organization of the EV-D68 capsid proteins. The canyon-like receptor-binding site is formed by VP1–VP3 on the viral surface, with VP4 nestled inside the particle, stabilizing the capsid structure.

**Figure 2:**
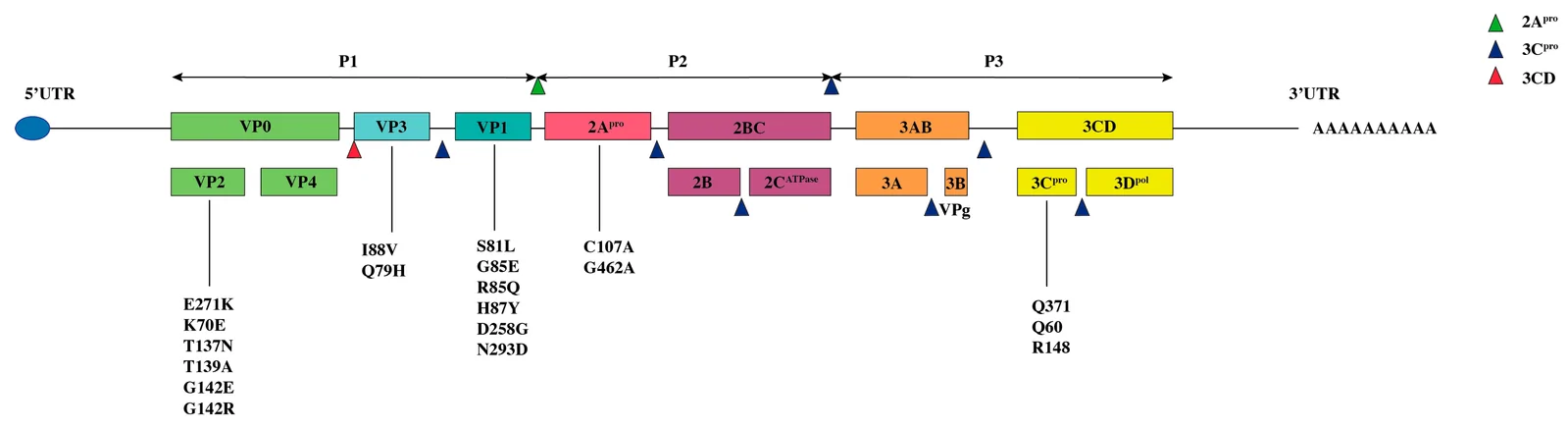
Proteolytic maturation and virulence site mapping of EV-D68 proteins. The schematic illustrates the cleavage of the P1 polyprotein into structural subunits VP1–VP4 and the organization of non-structural proteins P2 and P3, highlighting locations linked to pathogenicity. Potential mutation sites reported to influence pathogenicity are indicated.

EV-D68 is a single-stranded positive-sense RNA virus containing a single open reading frame (ORF) that encodes four structural proteins (VP1–VP4) and seven nonstructural proteins (2A–2C and 3A–3D) (Figure 1 and Figure 2). The structural proteins assemble to form the viral capsid, with VP1, VP2, and VP3 exposed on the virion surface, while VP4 is internally associated with the viral RNA. VP1 constitutes a major antigenic determinant recognized by the host immune system and is critically involved in receptor binding. Together with VP2 and VP3, it forms a surface “canyon” structure that facilitates specific attachment to host cell receptors and initiates viral entry [6].

The cellular tropism and pathogenesis of EV-D68 are defined by its use of multiple entry receptors. Its major identified receptors are sialic acid, heparan sulfate, ICAM-5/Telencephalin, and MFSD6. Sialic acid, present on the respiratory epithelium, facilitates initial attachment. Heparan sulfate enhances binding and promotes neurotropism, while the protein receptor MFSD6 mediates entry into respiratory and neuronal cells, modulated by the VP1 BC-loop. Unlike related enteroviruses such as EV-A71 (using SCARB2/PSGL-1) or CVA16 (SCARB2/KREMEN1), EV-D68’s use of sialic acid and MFSD6 defines its respiratory tropism. Furthermore, its capacity to bind heparan sulfate and the neuronal adhesion molecule ICAM-5 significantly contributes to its neurovirulent potential, a distinctive feature that sets EV-D68 apart from most other members of the enterovirus genus.

The nonstructural proteins coordinate various aspects of viral replication: 3D acts as the RNA-dependent RNA polymerase (RdRp), 2C and 3A contribute to the formation of replication complexes, and 2Apro and 3Cpro mediate polyprotein cleavage and host immune evasion.

The pathogenicity of EV-D68 is closely linked to specific genomic sequence elements that modulate viral replication, immune evasion, and host cell entry, thereby influencing clinical outcomes. This review summarizes current knowledge on the relationship between key sequence sites in the EV-D68 genome and viral pathogenicity, explores the underlying molecular mechanisms, and discusses the implications for surveillance and early response to future outbreaks.

## 2. Global Distribution of EV-D68 Subtypes

**Figure 3:**
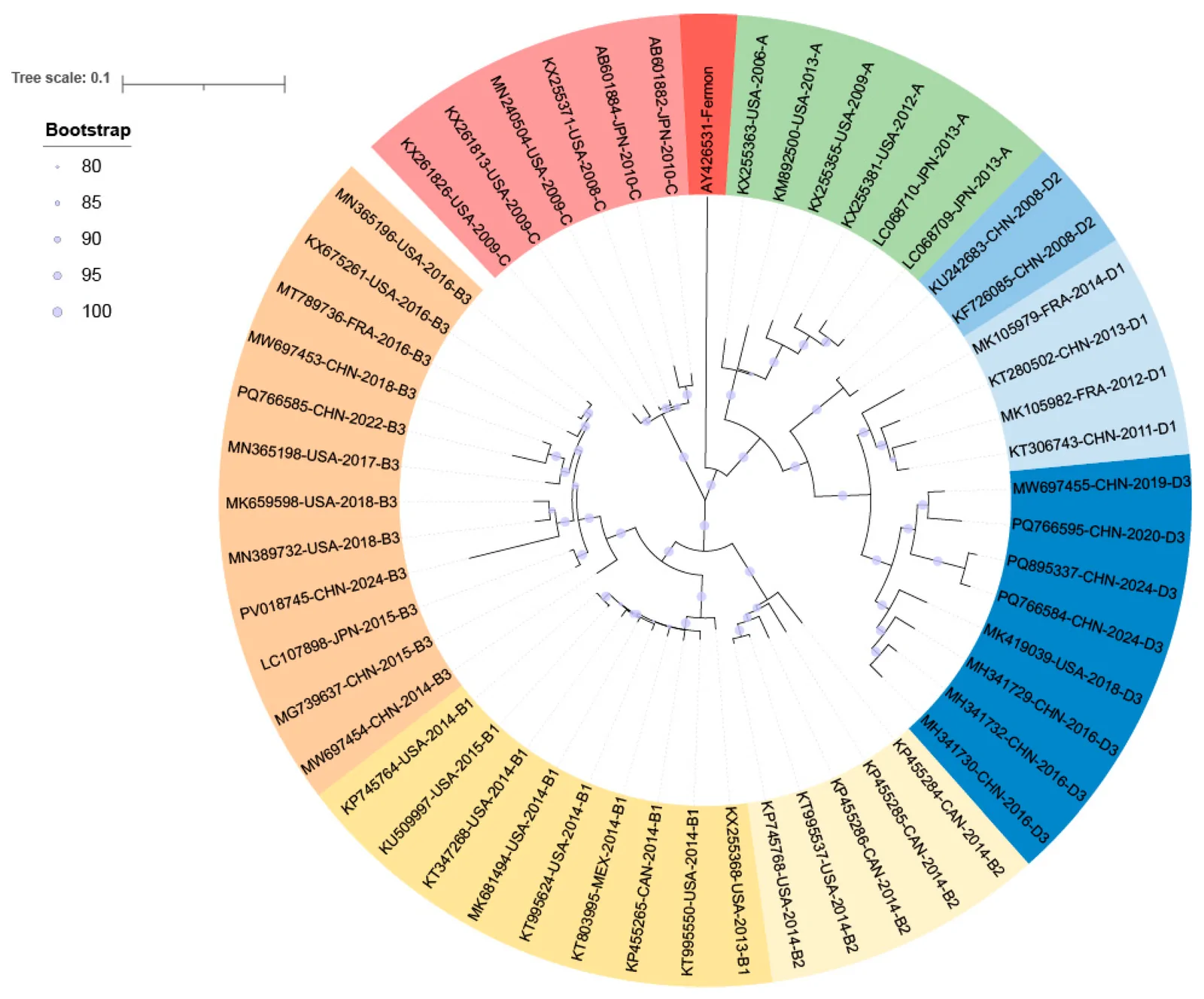
Phylogenetic analysis of global EV-D68 strains based on viral genome sequences. Different subtypes of the virus correspond to different colors.

EV-D68 exhibits a global distribution, with distinct temporal and geographic patterns in subtype prevalence and transmission dynamics. Current understanding of its epidemiology is likely incomplete, as EV-D68 infection is frequently underdiagnosed in clinical settings. This diagnostic gap stems in part from the variable sensitivity of available assays to detect the virus and differentiate between its genetic lineages. A phylogenetic tree constructed from representative strains of major subtypes illustrates this global diversity (Figure 3). Globally, subclade B3 has been the predominant lineage in recent years, while clade D (particularly D1) has been reported in Europe and the United States.

In North America, the United States experienced biennial epidemic peaks in 2014, 2016, and 2018. The 2014 outbreak was predominantly driven by subclade B1, with limited circulation of subclade B2. Subclade B3 first emerged as a predominant circulating lineage in 2016, while the D subtype was rarely detected in 2018 [7,8]. In Philadelphia, surveillance data from 2009 to 2018 revealed that the subclade B1 initially emerged in 2009, followed by annual epidemic peaks associated with AFM. By 2014, the subclade B1 had become the dominant lineage, whereas the B3 subtype persisted in circulation during 2016 and 2018 [9]. Meanwhile, environmental surveillance in 2021 revealed the co-circulation of clade D and subclade B3 [10]. In Canada, outbreak strains from 2014 primarily belonged to subclade B2 and were phylogenetically related to contemporaneous strains in the United States [11].

In Europe, subclade B3 was identified in Italy in 2016 and was genetically similar to strains circulating in southern China, the United States, and the Netherlands in 2015 [12]. In Spain during 2014–2016, subclades B2 and B3 were predominant, mirroring trends in the United States and other European countries. Subclade D1 emerged in 2017–2018 and was associated with pediatric cases presenting neurological symptoms, including meningitis and AFM [13]. In Finland, subclade B3 constituted the majority of strains detected in 2018 [14].

In Asia, widespread EV-D68 circulation in China was characterized by a shift in dominance from subclade A2 (2011–2013) to subclade B3 (2014–2018), with alternating detection of both lineages. Subclade B3 continued to circulate from 2017 to 2019, primarily causing mild acute lower respiratory tract infections in children, with rare reported neurological complications [15,16]. Surveillance in Shanghai from 2013 to 2020 identified the circulation of multiple subtypes, including D2, D3, and B3, albeit at low detection rates in children with respiratory tract infections. Subsequent sequence analysis suggested that recombination is a significant factor in shaping the viral genetic diversity [17]. Furthermore, subclade B1 and B3 were detected in Taiwan in 2008 and in India in 2016, respectively [18,19].

In South America, subclade B3 was first identified among children with respiratory infections in Brazil in 2017 [20]. Subsequent surveillance from 2021 to 2024 confirmed that B3 remains the dominant subtype in the country, alongside sporadic detections of D1 and D2 strains. In Africa, a study conducted in Senegal in 2016 identified EV-D68 in 7.4% (44/596) of patients presenting with influenza-like illness or acute flaccid paralysis, primarily affecting children under 5 years of age; all strains belonged to subclade B3 [21]. More recent surveillance data from Nigeria (2020–2022) and Kenya (2021–2023) further clarify the regional distribution. In Nigeria, subclade B3 co-circulated with a novel subclade designated B4, while in Kenya, both B3 and D1 strains were detected, with B3 comprising approximately 80% of isolates.

Recombination plays a crucial role in EV-D68 evolution, and co-circulation of different clades provides favorable conditions for recombination by increasing the likelihood of dual infection of a single host cell. For example, in Shanghai, the co-circulation of B3, D2, and D3 from 2013 to 2020 led to the emergence of B3/D3 recombinant strains. Similarly, in the United States, co-circulation of B1 and B3 in 2016 resulted in B1/B3 recombinants, which were found to have enhanced replication efficiency in respiratory epithelial cells. More recently, surveillance by the Johns Hopkins Medical System in Maryland identified a novel A2/B3 recombinant strain circulating during 2025. In accordance with standard virological nomenclature, such recombinant strains are designated by indicating their parental clades to denote their genetic origins.

## 3. Structural Protein-Related Sites and Pathogenicity

### 3.1. VP1 Region

VP1 is a core structural and functional component of the EV-D68 capsid, playing indispensable roles in viral infectivity, antigenicity, and pathogenesis. The VP1 gene spans approximately 927 nucleotides and encodes the principal antigenic epitopes recognized by the host immune system. These epitopes not only serve as the primary target for neutralizing antibodies but also provide the basis for EV-D68 serotype classification, enabling discrimination between viral variants [22,23] (Figure 2). Functionally, VP1 mediates viral receptor binding, immune evasion, and regulation of tissue tropism, making it the key structural protein influencing EV-D68 virulence.

Structurally, VP1 adopts a conserved β-barrel fold characteristic of enterovirus capsid proteins. Two surface-exposed loops—the BC-loop and DE-loop—are of particular functional importance. These loops are strategically positioned within the capsid architecture to facilitate two critical and often competing processes: interaction with host cell receptors and recognition by neutralizing antibodies [23]. This dual role renders them essential determinants of both infectivity and antigenic profile.

Studies have shown that specific amino acid residues within VP1, particularly in the BC-loop region, can modulate receptor binding affinity through conformational adjustments. For example, interactions with the host receptor MFSD6 are influenced by the structural flexibility of this domain [24,25]. Zhang et al., using neutralization escape mutant analysis, identified key residues in antigenic site I of the VP1 BC-loop—specifically G1085 and H1087 in the Fermon strain, and S1081 and R1085 in the clinical strain MO47—that map to the northern rim of the receptor-binding canyon. Mutations at these sites can alter sialic acid binding capacity and affect infection efficiency [26]. Additionally, the E271K substitution in VP1 has been shown to enhance viral replication in neuroblastoma (SK-N-SH) and rhabdomyosarcoma (RD) cell lines, potentially by strengthening heparan sulfate binding, which may promote spinal cord infection and neurovirulence [27]. Collectively, these site-specific alterations in VP1 represent important molecular markers associated with changes in viral pathogenicity.

### 3.2. VP2 and VP3 Regions

VP2 is one of the major surface-exposed capsid proteins in EV-D68, cooperating with VP1 and VP3 to maintain virion integrity and stability. Encoded by a gene of approximately 750–840 base pairs, VP2—along with VP3 (encoded by a 690–780 bp gene)—serves roles beyond structural support, contributing to virion assembly, immune modulation, and recognition by neutralizing antibodies [26] (Figure 2). These proteins jointly shape the surface topology of the capsid, including the conserved canyon region essential for receptor binding, thereby influencing both infectivity and immune evasion.

Zhang et al. identified that residues in the EF loop of VP2 (T2137, G2142) and the BC loop of VP3 (Q3079) constitute antigenic sites II and IV. Mutations at these sites may reduce capsid stability and enhance immune evasion. For example, the T2137N substitution in VP2 was shown to diminish neutralization sensitivity, potentially facilitating viral spread within the host [26]. Additionally, Chen et al. demonstrated that VP3 can suppress the interferon signaling pathway via interactions with host proteins [28]. Yeh et al. reported that the I88 V mutation in VP3 attenuates neurovirulence in mouse models, likely by altering VP3 conformation and impairing binding to neuronal surface receptors. A strain carrying this mutation (US/MO-14-18949) exhibited significantly reduced neurological pathogenicity [29]. Thus, VP2 and VP3, which are implicated in the formation of the surface canyon, serve as critical determinants of EV-D68 virulence.

In contrast to the external location of VP2 and VP3, VP4 is anchored to the inner surface of the capsid, where it interacts directly with the viral RNA to form a stable nucleocapsid core [26] (Figure 2). This internal positioning suggests a role in genome protection from nucleases and in maintaining RNA organization during assembly and maturation. Structural studies using cryo-electron microscopy have revealed that the C-terminal α-helix of VP4 inserts into the interface of capsid pentamers and forms a hydrogen-bond network with VP2 and VP3, reinforcing capsid stability throughout the viral life cycle. Currently, no virulence determinants have been conclusively assigned to VP4, likely due to limited direct functional studies and sparse sequence analysis across strains.

## 4. Association Between Non-Structural Protein-Related Sites and Virulence

### 4.1. A Protease

EV-D68 2A protease (2Apro) is a non-structural protein belonging to the trypsin-like cysteine protease family, defined by a conserved catalytic triad and stringent substrate specificity. Its proteolytic activity depends on three essential residues—His21, Asp39, and Cys107—which function cooperatively: Asp39 stabilizes the imidazole ring of His21, enabling deprotonation of Cys107 to generate a nucleophilic thiolate ion responsible for scissile bond cleavage. This structurally conserved active site ensures consistent 2Apro function across EV-D68 strains and underpins its central role in viral replication and pathogenesis [30,31].

Beyond catalyzing polyprotein processing, 2Apro acts as a key mediator of innate immune evasion. A major mechanism involves the specific cleavage of host tumor necrosis factor receptor-associated factor 3 (TRAF3), an adaptor protein essential for interferon (IFN) induction following viral RNA sensing. 2Apro recognizes and cleaves TRAF3 at glycine 462 (G462), a process strictly dependent on its own catalytic Cys107. Mutagenesis studies (e.g., C107A in 2Apro or G462A in TRAF3) confirm that ablation of this activity restores IFN production and attenuates viral evasion [30].

Furthermore, 2Apro collaborates with the viral 3C protease (3Cpro) to dismantle host translation machinery. Together, they target two central translation regulators: La-related protein 1 (LARP1), which facilitates the translation of 5’TOP motif-containing mRNAs, and poly(A)-binding protein cytoplasmic 1 (PABPC1), which stabilizes mRNA circularization and promotes translation initiation. Cleavage of these factors disrupts cap-dependent translation, thereby shutting down host protein synthesis and redirecting ribosomal resources to viral RNA [32]. This strategy not only cripples antiviral responses but also enhances viral protein production.

Although 2Apro mutations linked to virulence have so far been primarily associated with immune suppression, their multifaceted role in translational control implies broader contributions to pathogenicity that warrant further investigation.

### 4.2. C Protease

EV-D68 3C protease (3Cpro) is a multifunctional enzyme essential for viral replication, primarily responsible for cleaving the viral polyprotein into mature functional subunits. Beyond this canonical role, 3Cpro actively subverts host antiviral defenses through targeted cleavage of key immune-related host proteins. It further disrupts multiple cellular processes—including host translation, mitochondrial homeostasis, and inflammatory signaling—thereby reshaping the intracellular environment to favor viral persistence and propagation [33,34].

Structural studies indicate that the tertiary structure of 3Cpro varies among EV-D68 subtypes. For instance, the conformation of 3Cpro in Clade B1 differs from those in Clades A, B2, and B3, potentially influencing enzymatic efficiency and substrate specificity, which may contribute to differences in viral virulence [35]. Functionally, Wang et al. showed that 3Cpro cleaves LARP1 at Q371 and also targets PABPC1, thereby liberating viral RNA from translational suppression and impairing host mRNA stability and cap-dependent initiation [32]. In addition, 3Cpro cleaves the transcription factor TFEB at Q60, disrupting its interaction with Rag GTPases, which in turn impairs autophagy-lysosomal function and inhibits NF-κB activation. This attenuates inflammatory cytokine production and blunts the host antiviral response [36]. Li et al. reported that the host E3 ligase adaptor Cullin 3 restricts EV-D68 replication by promoting ubiquitination and degradation of 3Cpro. The K148 residue of 3Cpro was identified as a major ubiquitination site, while mutation at the adjacent R148 site impaired proteolytic activity [37].

Overall, 3Cpro appears to be critically involved in modulating virus-induced inflammatory pathways. Whether it also regulates cell death programs represents a promising direction for future investigation.

## 5. Untranslated Region Structure and Regulation of Viral Replication

The 3′-untranslated region (3′-UTR) of EV-D68 is a non-coding RNA segment located at the 3′ end of the viral genome that functions as a critical regulatory hub for viral replication and host–pathogen interactions. Unlike coding regions, its biological activity derives from conserved higher-order RNA structures—including stem-loops, pseudoknots, and bulges—that mediate sequence-specific molecular recognition [6]. These structural motifs contribute to genomic RNA stability and serve as docking platforms for host proteins, thereby bridging viral replication machinery with cellular pathways.

A study by Wang et al. elucidated a key mechanism by which the 3′-UTR subverts host antiviral defenses [38]. They identified an evolutionarily conserved element within the EV-D68 3′-UTR that specifically binds core stress granule (SG) proteins—TIA1, HUR, and G3BP1. Stress granules are dynamic ribonucleoprotein aggregates that sequester viral components to restrict replication. By recruiting these SG factors, the 3′-UTR disrupts granule assembly, rendering the cell permissive to viral propagation, and underscoring its role as a virulence determinant.

In a complementary study, Wang et al. employed a reverse genetics system to characterize the 5′-untranslated region (5′-UTR) of EV-D68 [39]. Through site-directed mutagenesis, they demonstrated that a single-nucleotide substitution (G394C) disrupts the secondary structure of the internal ribosomal entry site (IRES), impairing cap-independent translation initiation. This structural perturbation significantly reduced viral protein synthesis and replication, highlighting functional synergy between the 5′- and 3′-UTRs in sustaining the viral lifecycle.

The conserved sequence and structural features of both UTRs across EV-D68 isolates further suggest a role in viral pathogenicity. Structural conservation likely ensures reliable interaction with host factors and promotes efficient replication, which collectively influence infectivity and disease severity.

## 6. Virulence-Associated Loci in Other Enteroviruses

Many enteroviruses engage host receptors through defined capsid surface regions, such as the VP1 BC loop in EV-D68 and the VP1 N-terminus in EV-A71. Key residues in these regions, including K140 and R22, are conserved across multiple viral species, suggesting a shared strategy for receptor utilization [26]. For example, in Coxsackievirus A16 (CVA16), the VP1-N102D and VP1-E241K mutations are linked to viral attenuation [40]. Similarly, the VP1 T78A/G88A substitution in EV-A71 impairs proteolytic maturation of the VP0 precursor, reducing binding to the receptor SCARB2 and diminishing viral attachment and internalization, thereby attenuating virulence [41]. Moreover, certain distal capsid regions can modulate VP0 cleavage efficiency via conformational effects, influencing the yield of infectious virions—a mechanism conserved among multiple enteroviruses with differing virulence phenotypes [42].

Zhang et al. identified that VP2 residue K140 is invariant in all KREMEN1-dependent enterovirus A members, including CVA2, 3, 4, 5, 6, 10, and 12, and is essential for receptor binding, infectivity, and pathogenicity in mice [43]. Wang et al. reported that CVB3 exhibits polymorphism at VP3-234 (Q/N/V/D/E), with the Q234 variant conferring high-affinity binding to the CD55 receptor, potentially directing tissue tropism [44].

Beyond structural proteins, non-structural elements also modulate virulence. Peng et al. demonstrated that 2A proteases of EV-A71 and CVB3 undergo ERK-dependent phosphorylation at serine/threonine residues. A non-phosphorylatable mutation (S/T125A) impaired 2A protease activity, viral replication, and pathogenicity—a finding conserved across enteroviruses [31]. Zheng et al. showed that TRAF3 enhances STING-mediated antiviral responses against EV-A71, and that EV-D68 similarly activates the STING pathway but subverts it via 2A protease activity to facilitate immune evasion [45].

Variations in untranslated regions (UTRs) also contribute to virulence differences. In CVB3, structural divergence in the 5′ UTR of the avirulent CVB3/GA strain reduces translational efficiency and neurovirulence [46]. In CVA24v, specific 5′ UTR mutations are postulated to enhance transmissibility and virulence [47]. Furthermore, ubiquitination sites targeted by the host factor ZYG11B are conserved among CVA6, CVA16, and EV-D68; their modification suppresses viral replication, indicating a broad regulatory role in virulence [48].

Antigenic variation in VP1 underlies serotype-specific virulence in viruses such as EV-A71 and CVA16 [49]. Insertion–deletion (InDel) hotspots in Enterovirus A genomes also contribute to host recognition and immune evasion, further driving phenotypic diversification [50]. In Enterovirus B, specific virulence loci are associated with type 1 diabetes via islet autoimmunity [51], while CVB3 carries determinants linked to myocarditis and neurological sequelae [52]. Collectively, these findings underscore that virulence mechanisms identified in related enteroviruses may provide valuable insights for investigating pathogenicity determinants in EV-D68.

## 7. Literature Review of EV-D68 Virulence-Associated Sites

A systematic literature search was conducted using the Web of Science and PubMed databases for articles published between January 2010 and November 2025. Only English-language studies were included. The search identified 12 articles addressing virulence-associated sites in EV-D68. Of these, five investigated variations in structural proteins (VP1, VP2, and VP3), four focused on sites in the 2A protease and 3C protease, and two examined sequence or structural elements in the 5′-UTR and 3′-UTR (Table 1).

## 8. Challenges and Future Directions

The RdRp of EV-D68 plays an essential yet paradoxical role in viral evolution: although indispensable for genome replication, its lack of proofreading activity results in exceptionally high mutation rates. Unlike DNA polymerases, RdRp introduces nucleotide substitutions, insertions, and deletions at frequencies vastly exceeding those in host genomes. This genetic instability drives the continuous emergence of novel viral variants but complicates the identification of definitive virulence determinants, as phenotypic outcomes are often obscured by concurrent mutations across the genome [53].

EV-D68 pathogenicity is shaped by dynamic virus–host interactions, where demographic and immunological factors—such as young age, immunocompromised status, or pre-existing immunity—critically modulate severity. Capsid mutants that cause mild disease in immunologically mature hosts can lead to severe respiratory illness or AFM in immunologically naïve children. Similarly, interferon pathway deficiencies can worsen outcomes. Thus, virulence is not an intrinsic viral trait but emerges from the interplay of viral mutations and host susceptibility [54].

Moreover, changes in EV-D68 pathogenicity rarely result from single amino acid or nucleotide substitutions. Instead, virulence commonly arises from the cumulative effects of multiple sites distributed across viral proteins—such as VP1, 3Cpro, and RdRp—acting in concert. It is proposed that the clinical phenotype of EV-D68 infection is determined by the combined effects of multiple virulence determinants.

In light of these complexities, several key challenges impede progress. First, the predictive power of virulence-associated site identification remains limited: not all molecular changes observed in the laboratory correlate with clinical outcomes in humans. Second, while emerging tools—such as organoid models and artificial intelligence (AI)—show promise for early detection of high-risk variants, their translation into actionable public health interventions requires systematic validation and standardization.

To address these gaps, future efforts should prioritize the establishment of multidisciplinary surveillance networks that integrate genomic, clinical, and epidemiological data. Such systems will enable real-time tracking of viral evolution and enhance the early detection of emerging pathogenic strains. Equally important is the alignment of experimental models with clinical realities, ensuring that insights derived from cell cultures or animal studies are validated against human infection data. By closing the loop between bench and bedside, the field can move from reactive outbreak response to proactive risk prediction and preparedness.

## 9. Conclusions

In contrast to EV-A71, China has not experienced large-scale outbreaks of EV-D68. Moving forward, surveillance of viral virulence should prioritize key regions—including structural proteins VP1–VP3, proteases 2Apro and 3Cpro, and non-coding regions. Greater emphasis must be placed on correlating molecular findings with clinical phenotypes through rigorous validation. For both known and potential virulence loci, future virological studies should consider the combinatorial effects of multiple sites, rather than analyzing them in isolation, to better understand the polygenic nature of EV-D68 pathogenicity.

## Figures and Tables

**Table 1:** Summary of studies reporting virulence-associated sites in EV-D68.

Number	Year of Published	Virulence-Associated Sites	Genetic Region	Pathogenic Changes	Mechanism Classification	Research Platforms	Authors
1	2024	Novel peptide from VP1	VP1	Broad-spectrum antiviral activity against human enteroviruses	Immune modulation	Cell lines	Lin, X.; et al. [22]
2	2015	N	VP1	Structure and inhibition of EV-D68	Receptor binding regulation, Inhibition target	Structural analysis	Liu, Y.; et al. [23]
3	2025	MFSD6	VP1	Reducing virus replication	Receptor binding regulation	Mouse model and Cell lines	Liu, X.; et al. [24]
4	2023	S1081L, G1085E, R1085Q, H1087Y, D1285G, N1293D, K2070E, T2137N, T2139A, G2142E, G2142R, Q3079H	VP1, VP2, VP3	Inhibiting viral binding to cells	Receptor binding regulation, Immune escape	Cell lines	Dai, W.; et al. [26]
5	2020	E271K	VP1, VP2	Resulting in a decrease in attachment, internalization, and replication of viruses	Receptor binding regulation, Immune escape	Cell lines	Sooksawasdi, N.A.S.; et al. [27]
6	2025	VP3 co-localizes	VP3	Facilitating immune evasion and promoting viral replication	Immune escape	Cell lines	Chen, H.; et al. [28]
7	2020	I88V	VP3	Reducing virus replication	Receptor binding regulation	Mouse model and Cell lines	Yeh, M.T.; et al. [29]
8	2021	TRAF3	2Apro	Facilitating subversion of host innate immune responses	Immune escape	Cell lines	Kang, J.; et al. [30]
9	2025	N	2Apro, 3Cpro	Inhibiting EV-D68 replication and reducing the virus-mediated suppression of host translation	Translation regulation	Cell lines	Tan, R.; et al. [32]
10	2015	N	2Apro, 3Cpro	Altering the proteases’ cleavage efficiency, leading to increased rate of viral replication and transmission	Protease activity regulation	Whole-Genome sequence analysis	Huang, W.; et al. [35]
11	2024	TFEB	3Cpro	Inhibiting autophagic flux and promotes virus egress	Autophagy regulation	Cell lines	Jassey, A.; et al. [36]
12	2025	R148	3Cpro	Restricting the replication of the virus	Protease activity regulation	Mouse model and Cell lines	Li, Y.; et al. [37]
13	2020	SG	3′-UTR	Inhibiting viral replication	Replication regulation	Cell lines	Cheng, J.; et al. [38]
14	2018	N	5′-UTR	Suppressing the viral cap-independent translation	Translation regulation	Cell lines	Pan, M.; et al. [39]

## Data Availability

No new data were created or analyzed in this study. Data sharing is not applicable to this article.

## Abbreviations

The following abbreviations are used in this manuscript:

EV-D68	Enterovirus D68
AFM	Acute flaccid myelitis
RdRp	RNA-dependent RNA polymerase
bp	Base pairs
2Apro	2A protease
TRAF3	Tumor necrosis factor receptor-associated factor 3
LARP1	La-related protein 1
PABPC1	Poly A-binding protein cytoplasmic 1
3Cpro	3C protease
3′-UTR	3′-untranslated region
5′-UTR	5′-untranslated region
SGs	Stress granules
EV-A71	Enterovirus A71
CVB3	Coxsackievirus B3
CVA	Coxsackievirus A
InDel	Insertion-deletion
AI	artificial intelligence
