# Correction: Zhu et al. Enterovirus D68 Sequence Variations and Pathogenicity: A Review. *Viruses* 2026, *18*, 73

**DOI:** 10.3390/v18070796

**Published:** 2026-07-20

**Authors:** Yi Zhu, Liting Wang, Jun Shen

**Affiliations:** Infectious Disease Department, Children’s Hospital of Fudan University, National Children’s Medical Center, Shanghai 201102, China

**Missing Citation**

In the original publication [1], the following reference was not cited:

Varanese, L.; Xu, L.; Peters, C.E.; Pintilie, G.; Roberts, D.S.; Raj, S.; Liu, M.; Ooi, Y.S.; Diep, J.; Qiao, W.; et al. MFSD6 is an entry receptor for enterovirus D68. *Nature* **2025**, *641*, 1268–1275. https://doi.org/10.1038/s41586-025-08908-0.

To ensure the integrity of the literature review, this reference has now been included as reference [25] in the reference list. Consequently, a citation has been inserted in 3.1. VP1 Region and should read:

Studies have shown that specific amino acid residues within VP1, particularly in the BC-loop region, can modulate receptor binding affinity through conformational adjustments. For example, interactions with the host receptor MFSD6 are influenced by the structural flexibility of this domain [24,25]. Zhang et al., using neutralization escape mutant analysis, identified key residues in antigenic site I of the VP1 BC-loop—specifically G1085 and H1087 in the Fermon strain, and S1081 and R1085 in the clinical strain MO47—that map to the northern rim of the receptor-binding canyon. Mutations at these sites can alter sialic acid binding capacity and affect infection efficiency [26]. Additionally, the E271K substitution in VP1 has been shown to enhance viral replication in neuroblastoma (SK-N-SH) and rhabdomyosarcoma (RD) cell lines, potentially by strengthening heparan sulfate binding, which may promote spinal cord infection and neurovirulence [27]. Collectively, these site-specific alterations in VP1 represent important molecular markers associated with changes in viral pathogenicity.

With this correction, the order of some references has been adjusted accordingly. The authors state that the scientific conclusions are unaffected. This correction was approved by the Academic Editor. The original publication has also been updated.
